# The “Gum–Gut” Axis in Inflammatory Bowel Diseases: A Hypothesis-Driven Review of Associations and Advances

**DOI:** 10.3389/fimmu.2021.620124

**Published:** 2021-02-19

**Authors:** Kevin M. Byrd, Ajay S. Gulati

**Affiliations:** ^1^ Division of Oral & Craniofacial Health Sciences, University of North Carolina Adams School of Dentistry, Chapel Hill, NC, United States; ^2^ Department of Innovation & Technology Research, ADA Science & Research Institute, Gaithersburg, MD, United States; ^3^ Division of Gastroenterology, Department of Pediatrics, University of North Carolina at Chapel Hill, Chapel Hill, NC, United States; ^4^ Department of Pathology and Laboratory Medicine, University of North Carolina at Chapel Hill, Chapel Hill, NC, United States

**Keywords:** gum–gut, oral–gut, microbiome, gingivitis, periodontitis, Crohn’s disease, ulcerative colitis, inflammatory bowel disease

## Abstract

In modern medicine, the oral cavity has often been viewed as a passive conduit to the upper airways and gastrointestinal tract; however, its connection to the rest of the body has been increasingly explored over the last 40 years. For several diseases, the periodontium and gingiva are at the center of this oral-systemic link. Over 50 systemic conditions have been specifically associated with gingival and periodontal inflammation, including inflammatory bowel diseases (IBD), which have recently been elevated from simple “associations” to elegant, mechanistic investigations. IBD and periodontitis have been reported to impact each other**’**s progression *via* a bidirectional relationship whereby chronic oral or intestinal inflammation can impact the other; however, the precise mechanisms for how this occurs remain unclear. Classically, the etiology of gingival inflammation (gingivitis) is oral microbial dysbiosis in the subgingival crevice that can lead to destructive periodontal disease (periodontitis); however, the current understanding of gingival involvement in IBD is that it may represent a separate disease entity from classical gingivitis, arising from mechanisms related to systemic inflammatory activation of niche-resident immune cells. Synthesizing available evidence, we hypothesize that once established, IBD can be driven by microbiomial and inflammatory changes originating specifically from the gingival niche through saliva, thereby worsening IBD outcomes and thus perpetuating a vicious cycle. In this review, we introduce the concept of the “gum–gut axis” as a framework for examining this reciprocal relationship between the periodontium and the gastrointestinal tract. To support and explore this gum–gut axis, we 1) provide a narrative review of historical studies reporting gingival and periodontal manifestations in IBD, 2) describe the current understanding and advances for the gum–gut axis, and 3) underscore the importance of collaborative treatment and research plans between oral and GI practitioners to benefit this patient population.

## Introduction

### A Bidirectional Influence of Oral and Systemic Health

The oral cavity serves as the entry point to the gastrointestinal tract and is also continuous with the nasal cavity and the skin of the face (1). While it certainly functions as a conduit for the movement of food, fluids, and air, this space has been revealed to be a diverse collection of tissues that are harmoniously integrated into the vital functions of communication, defense, feeding, breathing, and early digestion (2–4). Diseases of these tissues range from innocuous, seriously disabling, or even lethal. Indeed, they were recognized as such by Hippocrates, who cataloged oral diseases as part of—not separate from—the whole body (5, 6). Despite this ancient perspective and recent efforts by healthcare leaders to breakdown longstanding barriers, the concept of oral medicine existing separate from general medicine persists (7–9).

**Figure 1:**
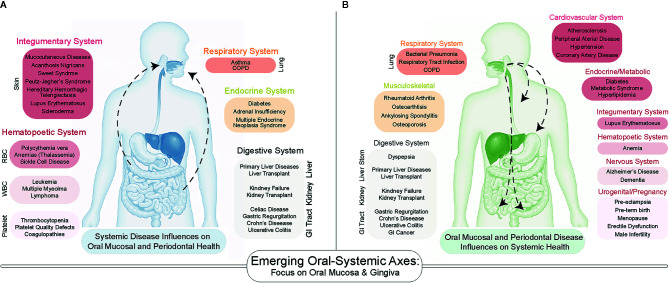
A Bidirectional Influence of oral and systemic health. **(A)** Emerging associations between systemic disease on oral health include diseases of the skin, lungs, gastrointestinal tract as well as endocrine and hematopoietic systems. **(B)** The number of systemic diseases impacted by oral inflammatory diseases of the periodontium (gingivitis and periodontitis) continues to increase as more studies are conducted. Many of the same systems that influence oral health are influenced by periodontal health.

While many pathologies are confined to the oral cavity itself, there has been increasing exploration of the links between oral diseases and systemic health. We and others hypothesize that this is a bidirectional link, centered around the generalized influence of chronic inflammation. Specifically, there exist several oral-systemic axes in which inflammatory diseases of the oral cavity can lead to dysbiosis, which then influences the systemic disease course—and vice versa. For example, numerous systemic diseases demonstrate manifestations in the oral cavity (**Figure 1A**). In particular, several nutritional deficiencies and systemic diseases involving the skin, hematopoietic system, immune system, endocrine system, connective tissues, lungs, liver, kidneys, and the gastrointestinal tract are known to demonstrate a diverse array of bony, glandular, connective tissue, and mucosal manifestations in the oral cavity (10, 11). While the purpose of this review is not to catalog all known or suspected oral-systemic axes, it is evident that these links are likely underappreciated in both oral health care and in medicine.

The mechanistic underpinnings for each of these oral-systemic axes are limited. The reasons for this are numerous, but one important consideration is that they can present unpredictably and are known to present in specific oral niches. The soft tissues of the oral cavity are heterogeneous, comprised of transitions between masticatory and lining mucosal tissues, taste and tactile papillae on the dorsal tongue, major and minor salivary glands, palatine and lingual tonsillar tissues, and the tooth and its supporting periodontium—comprised of the periodontal ligament, cementum, alveolar bone, and the gingiva (12). When considering the involvement of systemic disease in the oral cavity, the uniqueness of these oral tissues inadvertently predilects some sites to be more or less likely to display the influence of systemic effects. One of the best examples of this is in inflammatory bowel diseases (IBD), which can present in nearly every niche, including the lymph nodes, buccal mucosa (cheek lining), tongue, lips, teeth, and periodontium (13). The most common oral manifestations of IBD involve the buccal mucosa and the gingiva, both of which can display severe, chronic inflammatory lesions. This is not surprising, as the anatomy of the periodontium makes this a susceptible site for frequent dysbiosis and chronic inflammation.

Among all oral niches, the periodontium has been the most often explored as key to the oral-systemic link (14, 15), and the influence of chronic inflammatory diseases of the periodontium (gingivitis and periodontitis) on systemic healthy was formalized as the term “periodontal medicine” to describe these gum-systemic links in the 1990s (16–20). To date, over 50 diseases have been associated with gingivitis/periodontitis (21), including atherosclerotic disease (22), adverse pregnancy outcomes (23), type I and type II diabetes (24, 25), metabolic syndrome (26), and inflammatory bowel diseases (**Figure 1B**) (27). The directionality and causality of these associations remain to be elucidated, but recent mechanistic investigations into the oral-systemic link in IBD have recently provide clues into the interworking of this axis (28, 29). This review will focus on the periodontium as a specialized tissue niche that not only displays involvement in IBD but may also be able to seed those local changes to the distant gut to exacerbate the course of IBD. Throughout this review, we will synthesize what is known across many fields to provide support for an emerging oral–gut link in IBD. However, we will emphasize the idea of this phenomenon likely being uniquely associated with gingival and periodontal inflammation and thus, we introduce the concept of the “gum–gut axis” for the first time as a framework for examining the reciprocal relationship between the periodontium and the gastrointestinal tract (i.e., gut-to-gum influences and gum-to-gut influences). To support and explore this emerging gum–gut axis, we 1) provide a narrative review of historical studies reporting gingival and periodontal manifestations in IBD, 2) describe the current understanding and advances for the gum–gut axis, and 3) underscore the importance of collaborative treatment and research plans between oral and GI scientists to benefit this patient population.

## Background

### Periodontitis and Periodontal Disease as a Manifestation of IBD

Currently, about 40% of all US adults older than 30 have some form of periodontal disease (30), and ~11% of the world**’**s population is currently diagnosed with a severe form of the disease (31). Periodontal diseases are immune-mediated, chronic disorders of the periodontium. Based on the 2017 World Workshop on the Classification of Periodontal and Peri-Implant Diseases and Conditions, diseases of these tooth-supporting tissues are now classified into gingivitis, as well as three major periodontal disease categories: (a) periodontitis, (b) necrotizing periodontal diseases, and (c) periodontitis as a manifestation of systemic disease (32). Periodontal diseases typically originate in the gingiva as inflammation before causing progressive alveolar bone destruction (33, 34). Over many years of work, it is known that gingivitis and periodontitis are caused by a shift from a healthy to a dysbiotic biofilm in the subgingival crevice or “pocket” (35, 36). Once established, periodontal diseases display extensive disease heterogeneity but are commonly defined by chronic and destructive periodontal inflammation that can lead to loss of tooth-supporting tissues and a lower quality of life (37). These diseases are often diagnosed after 30 years old (38); however, it is also important to note that severe gingivitis can occur at any age, even in children.

Similarly, IBD represents a group of immune-mediated, chronic inflammatory disorders of the gastrointestinal tract. They are typically characterized into two primary disease types: Crohn**’**s disease (CD) and ulcerative colitis (UC). Among these diseases, there is signifcant heterogeneity within these subtypes, and many more IBD phenotypes likely exist than have been defined. The incidence of IBD in the US and Europe has recently stabilized (39, 40), yet the US still has about 25% of the world**’**s cases when age-standardized metrics are utilized (40). Pediatric IBD comprises about 25% of all cases, and these patients typically have more aggressive disease, with up to 34% requiring surgery within 10 years of diagnosis (41, 42). Modern twin studies suggest the heritability of both CD and periodontitis is around 0.3, though slightly less for UC (43, 44). Both IBD and periodontitis are actively being investigated for perturbations of host genetics, the microbiome, environment, diet, and inflammatory/immune cell subtypes as potentially explaining disease progression (45, 46).

### Gingival and Periodontal Manifestations of IBD

While the extraintestinal manifestations of IBD may involve the skin, eyes, and joints (47, 48), oral involvement can occur in up to 50% of all cases. For pediatric IBD, oral manifestations— often in the gingiva—may be present as high as 80%, with higher prevalence in males and CD (49–52). Though reports vary, it has been suggested that up to 25% of IBD cases present with oral symptoms before any intestinal involvement (48, 53); that said, the most frequently reported oral manifestations are the appearance of “cobblestoning” and ulceration of the oral mucosa (13) as well as chronic, severe inflammation of the gingiva/periodontium (54). It is noteworthy that while IBD patients commonly display severe gingival inflammation and hypertrophy, lesions of the gingiva can also be subtle. For example, mild inflammation of the gingival margin may present as a subclinical lesion (marginal gingivitis) (55); moreover, there is significant heterogeneity of gingival lesions generally, despite controlling for similar patient demographics (56). Even among trained providers, diagnosing gingival disease can be challenging and time-consuming (49, 57, 58); thus, it is likely that the prevalence of gingival manifestations of IBD at a “person-level” is likely underreported. To explore the gum–gut axis in IBD, it is imperative that we first explore the pathogenesis of gingivitis and compare it to what is known about gingivitis in IBD.

### Classical Gingivitis *vs* IBD-Induced Gingivitis

**Figure 2:**
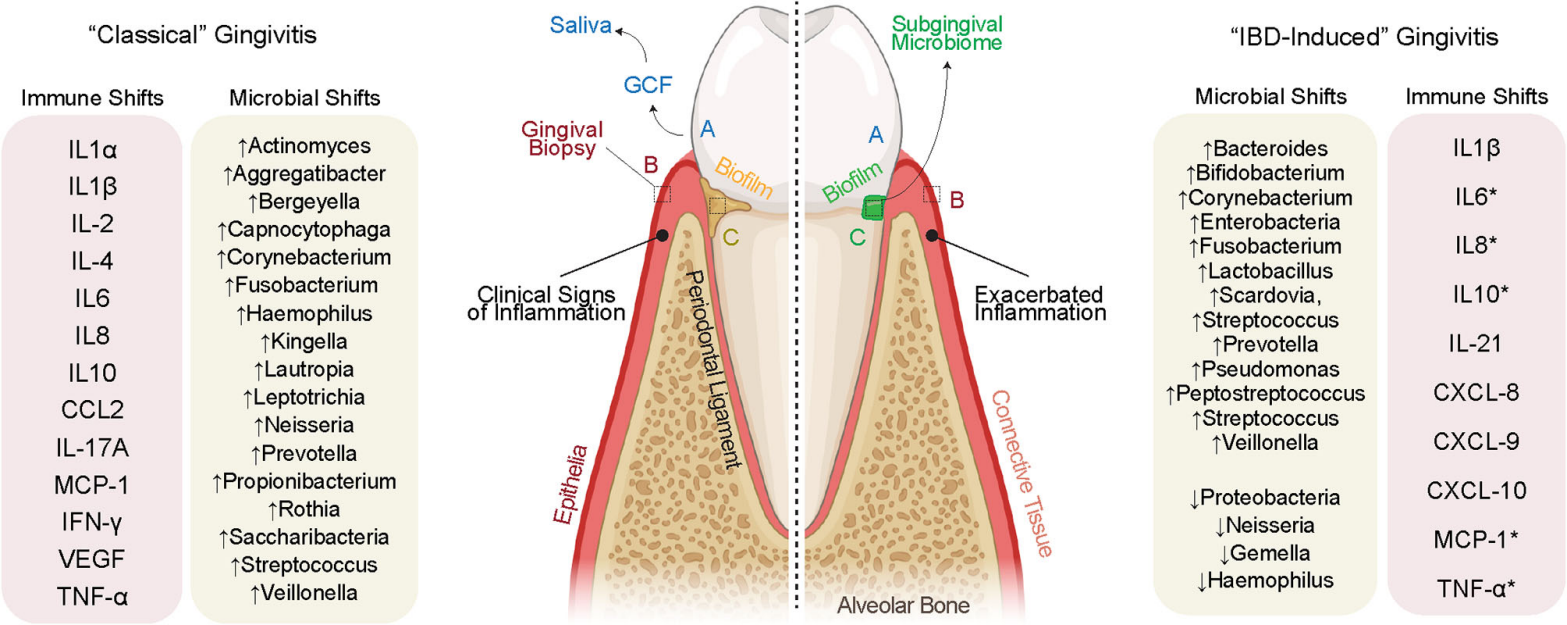
Evidence for IBD-induced gingivitis as a separate disease entity. (Left) “Classically” defined gingivitis involves well-defined increases to pro-inflammatory cytokines secondary to defined shifts in the microbiome. The inflammation clinically documented in IBD often discordant with biofilm deposits on the tooth surface, suggesting a role for the systemic inflammation of IBD to cause oral inflammation in parallel with or independent of biofilm deposits. This inflammation may itself shift the oral microbiome which then may cause further gingival inflammation. A sampling of the gingival crevicular fluid (GCF) and saliva (biofluids), gingival tissues (full-thickness biopsies), and subgingival microbes (microbiome) allows for a detailed understanding of the inflammatory and microbiomial shifts shared and unique to these possibly unique diseases.

In health, gingivae appear pink in color, firm to palpation, and occasionally stippled with no obvious pathology (i.e., bone resorption; **Figure 2**) (59). Diseases of the gingiva are often associated with inflammation of the gums/gingiva, and by definition, involve only the soft tissues of the periodontal attachment (60). Like periodontitis, it has been shown that gingivitis is caused by local dysbiosis of the subgingival crevice (i.e., in the subgingival microbiome) (61, 62); however, the gingival inflammation observed in IBD patients does not appear to follow this well-known pathogenesis in all cases. Extracting from recent reports, it appears that gingival and periodontal inflammation in IBD may not be biofilm-induced, but rather, biofilm-exacerbated (27). This would establish a paradigm for the **gum–gut** axis whereby the treatment modalities and understanding of the disease cascade may not necessarily follow these classic studies and raises the possibility that IBD-induced gingivitis could be a different disease entity altogether. This requires further exploration.

In classical gingivitis (or simply “gingivitis” for this review), inflammation of the gingival margin presents with erythema as well as increases in the inflammatory infiltrate (63). Understanding the differences between gingivitis and IBD-induced gingivitis pathogenesis will be important but also challenging. This is because gingivitis is simultaneously reversible and increasingly prevalent as we age; ~1/3 of all 3-year-olds, ~2/3 of 5-year-olds, and >90% of young adults have gingivitis (64). Gingival and periodontal diseases can present from to mild to severe, often historically classified as initial, early, established, and advanced lesions (62). The “initial” lesion of gingivitis is not detectable clinically but has been shown to occur within 2–4 days after biofilm accumulation. This results in an increase in gingival crevicular fluid flow, vasoactive compound release, neutrophil migration into the gingival crevice, and finally the release of effector cytokines to induce more inflammation (59). Through progressive changes, the classical “early” lesion is established within a week of biofilm accumulation and is defined by an increased relative abundance of lymphocytes and macrophages (65). While the response of the host tissues is increasingly heterogeneous when examining individuals (66), the more mature established gingival lesion is defined by a significant increase in B and plasma cells (59).

In gingivitis, the subgingival microbiome undergoes a measurable shift in taxa that elicit an immune response [see list in **Figure 2**; (67)]. While these species are found in the subgingival microbiome, the host inflammatory response in gingivitis is now thought to be a result of alterations to microbiomial abundance, richness, and interspecies interaction (67). While there is important person-to-person variation, the result of sustained gingivitis in susceptible individuals is periodontitis, often defined by an increased presence of *Porphyromonas gingivalis*, *Tannerella forsythia*, *Treponema denticola*, and *Aggregatibacter actinomycetemcomitans* (68). While these concepts of microbiomial “ecology” are still being explored in gingivitis, the similarities and differences for IBD-induced gingivitis are only now emerging after years of case reports and association studies.

## The History of the “Gum–Gut” Axis in IBD

### Classical Case Reports

**Figure 3:**
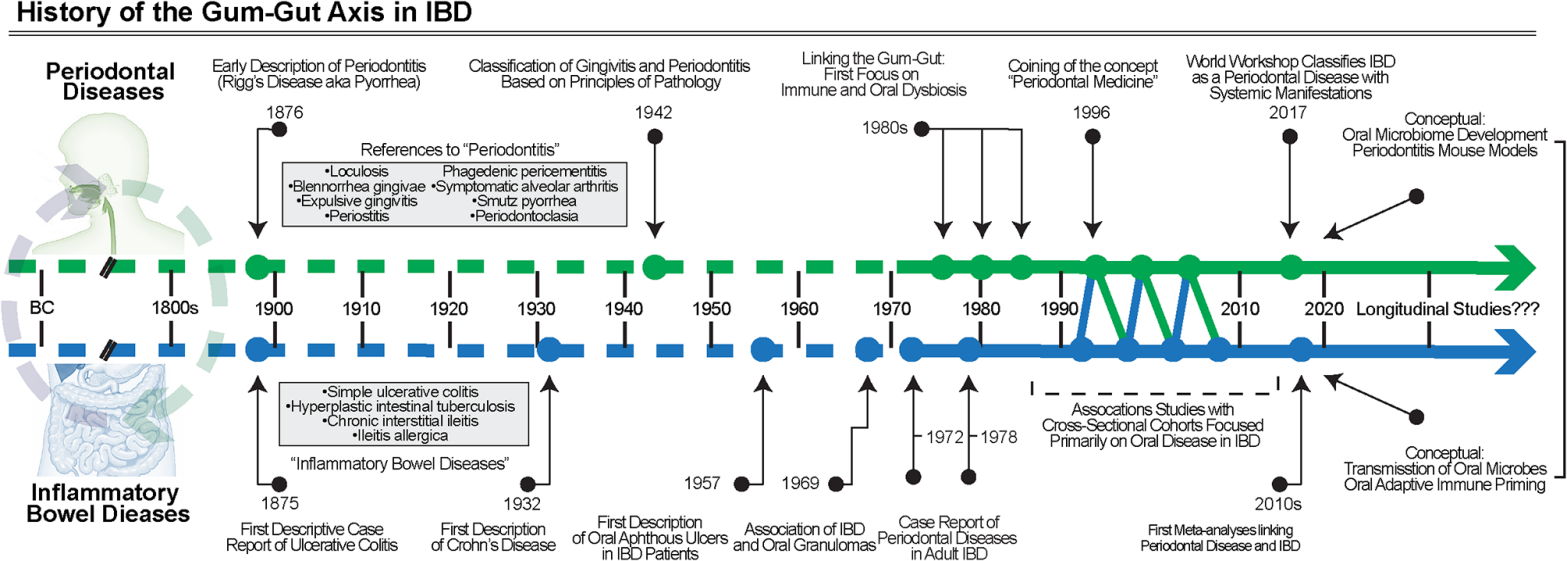
The History of the gum–gut axis in IBD. Over many centuries, the chronic inflammatory diseases of the gut and periodontium were noted but not classified into defined disease entities until the late 1800s and early 1900s. First with case reports, then association studies, and finally clinical trials using biosampling primarily of the oral cavity, the evidence for the bidirectional relationship became better understood. More studies are required, including longitudinal cohorts, to understand the temporal associations and to further test causality.

Like periodontal diseases, the signs and symptoms of IBD have been alluded to throughout human history. UC was first described in case series from the late 19^th^ century, and separately, CD in 1932 (69, 70). Periodontal disease classifications have been dynamically revised over the years and multiple classifications have been proposed, starting as early as the late 19^th^ century; however, it was not until 1942 that a classification paradigm was based on the principles of pathology detailed “gingivitis” or “periodontitis” (7, 71). As early classifications of these diseases became better understood and more widely disseminated, only then were studies of gingival and periodontal manifestations in IBD even possible to conduct (**Figure 3**).

Oral manifestations of IBD were first reported in the 1950s and initially focused on aphthous ulceration (72), though many reviews reference a description of oral granulomatous inflammation from Dudeney and Todd in 1969 as the first report of oral involvement in IBD (73). Over the following decades, detailed case reports were published that highlighted the diversity of oral manifestations in IBD, generally highlighting the most severe lesions (74–77). For example, one early case description detailed an account of a pediatric CD patient who developed unilateral granulomatous inflammation in the buccal mucosa 6 years after the initial diagnosis (73). Over the next few years, similar case reports were published expanding these observations of oral manifestations in IBD patients to soon detail other sites such as the lips and the hard palate (75, 78, 79).

Among these studies, gingival and periodontal manifestations of IBD were also reported in the literature, often including patients presenting with severe gingivitis (73, 80, 81). In these studies, similarities were noted between the pathogenesis of periodontitis and IBD. The connection between IBD and specific manifestations in the gingiva was documented around the same time as other manifestations. For example, a case report from 1972 detailed findings from a pediatric CD patient who presented with gingival hyperplasia on the entire maxillary anterior teeth (80). The tissues were described as 5–6 mm “pseudopockets”, suggestive of a severe hyperplastic phenotype that worsened periodically. While there was no bony involvement—and therefore not truly periodontitis—this was a unique manifestation of IBD and suggested that chronic, severe gingivitis in IBD patients may itself undergo temporal exacerbation and remission. As early as 1978, the first studies suggesting severe periodontitis might be associated with IBD, which were published (82–84). These reports provided some of the earliest evidence for a “gum–gut” axis in IBD and highlighted gaps in knowledge related to the long-term impact of this chronic periodontal inflammation over the life span.

### Association Studies

Building on these early case reports, cross-sectional studies were subsequently designed to search for positive associations among IBD and periodontal afflicted individuals. Some of the earliest studies on these gum–gut associations focused on the gingiva of children. A later study specifically assessed gingival inflammation in children and adolescents (aged 4–18 years old) (85). Interestingly, this report focused on a remission cohort of patients receiving treatment with immune-modulating medications (i.e., anti-TNF; others). Even though the matched healthy controls and IBD subjects displayed similar oral health habits, IBD patients self-reported a significantly higher incidence of bleeding gums when brushing and had higher gingival inflammation scores upon clinical examination. Based on the Community Periodontal Index of Treatment Needs (CPITN) index, none of the IBD patients were determined to have healthy periodontal tissues (0/55 subjects), and about 2/3 had a high need for periodontal treatment (scores >1). This highlights that the use of clinical indices of periodontal disease severity can better define the strength of the gum–gut axis.

Studies like these presented substantial support for a gum–gut axis in which gingival inflammation was a primary manifestation of IBD, even in children. Ultimately, longitudinal studies will be required to truly understand the impact of early gum inflammation on the gut in the long-term. Because such prospective cohort studies are yet to be conducted, most association studies have focused on adult cross-sectional populations. However, few of these studies have documented critical variables such as the length of time since IBD diagnosis or measures such as the severity over time, which does lead to some difficulty in interpreting results. For example, a 1991 study showed that IBD patients display a higher prevalence—but a decreased severity—of periodontitis (86). A 2006 report assessed periodontitis in patients diagnosed with IBD using a case-control study design and again showed a trend toward lower severity, but higher periodontal disease prevalence, in IBD patients (87). These unexpected findings support the idea that that gingivitis and IBD-induced gingivitis are unique and underscore that while IBD-induced gingival inflammation is often documented to be more clinically severe, this mechanism of inflammation may be less likely to lead to the tissue destruction seen in periodontitis (88, 89). This is at odds with what is known for the classical dysbiotic-driven disease course and suggests more is left to learn for both IBD and healthy individuals.

Other studies have also found consistent positive associations between periodontitis and IBD compared to healthy controls, using standard indices of periodontal inflammation in adults (88, 89). For example, a 2013 article detailed 113 patients with IBD compared to healthy controls and found all clinical markers of periodontitis such as bleeding on probing, loss of clinical attachment, and pocket depth were each increased in both UC and CD patients (90). Additional case-control studies have demonstrated that IBD is positively associated with common clinical indices of gingivitis and periodontitis and that moderate-to-severe CD activity correlates with clinical indices of periodontal disease (90). A recent meta-analysis has aggregated these cohorts and summarized the results of six studies (total: 599 IBD patients and 448 control subjects), revealing significant positive risk associations for periodontitis in CD (3.64; 95% CI: 2.33–5.67) and UC (5.37; 95% CI: 3.30–8.74) (91). Another meta-analysis of 9 cross-sectional studies similarly found risk ratios of 4.55 for having periodontitis in IBD patients (92). These documented associations of periodontitis and IBD may be established long before periodontitis manifests, and when considering the mechanisms that drive the gum–gut axis, there is an unmet need to understand how IBD-induced periodontal manifestations are compared to classically understood concepts in IBD, including tissue barrier, inflammation, and microbiomial dysbiosis.

## Factors that Drive the Gum–Gut Axis

### Gingival Barrier

The gingiva, which is comprised of oral epithelium, connective tissue, blood and lymphatic vessels, smooth muscle, and fibroblasts, forms around each tooth as it erupts. The overlying oral epithelia fuse with the specialized, tooth-associated reduced enamel epithelium, the latter which establishes the soft tissue attachments to the non-shedding surfaces of the tooth *via* hemidesmosomes. This developmental process transforms the stratified squamous epithelia into both a sulcular epithelium and tooth-associated junctional epithelia over 4 years to form the gingival sulcus—and physical barrier—circumferentially around each primary and permanent tooth (59, 93, 94). It is at this critical niche where a dynamic host structural, host immune, and microbial relationship influences disease initiation and progression. Despite its innate and acquired immune defense, it remains incredibly susceptible to disease. These host tissues, however, are also increasingly considered an immune organ, performing functions necessary to withstand assaults from the mechanical forces associated with mastication and from frequent microbiomial shifts (95, 96). Even in health, more inflammatory cells are detected at the gingival barrier compared to other oral sites, suggesting this site may be able to readily attract immune cells to mount a defense against a shifting subgingival microbiome (97, 98).

This barrier site displays an underappreciated and poorly understood epithelial and mesenchymal cell heterogeneity (99, 100) but has an incredible ability to tolerate the stress of the oral environment, to regenerate after periodontal surgeries (94), and has an emerging role in immune cell recruitment and “crosstalk” (101, 102). This makes this niche one of the most dynamic in the oral cavity. These vulnerable cells are held together by cell-cell adhesions such as cadherins (CH1, CDH3), desmogleins (DSG1, DSG2, DSG3), desmocollins (DSC1, DSC2, DSC3), and tight junctions (TJP1, OCLN, CLDN1, CLDN7, CLDN10, CLDN12) (103, 104). The expression of these adhesion genes is heterogeneous by gingival epithelial cell type, which is of interest considering recent work that suggests the fine-tuning of immunity occurs from the structural cells present in various body niches (38). Also of relevance is the impact of diet on the host barriers in the oral cavity and intestine, which suggest vitamins C primarily from fruits may play a potential protective effect across the gastrointestinal tract (105–108). There is much still to be learned about the impact of the environment, diet, and niche-specific immune interactions in periodontal diseases. A broader and deeper understanding of these modifiers will help us understand how gingival inflammation in IBD is related to gingivitis. However, studies to date have only focused on host inflammation and oral microbiome in IBD.

### Systemic Inflammation

Some of the earliest studies that considered the mechanisms of the gum–gut axis include reports in the late 1970s/1980s from Lamster et al. and Engel et al. which suggested functional changes to the immune system may play a common and important role in patients with CD and periodontitis pathogenesis (83, 84, 109). These studies provide a framework on how this nascent field would approach studies of the gum–gut axis in IBD: through profiling the inflammatory milieu, sampling the oral microbiome, or both; however, this association is not causality, even considering more sensitive methods of sampling and profiling.

For convenience, saliva has often been collected as a surrogate for localized tissue inflammation, though profiling of the gingival crevicular fluid around the inflamed gingiva would be assumed to provide a clearer signal. Despite this, salivary sampling in CD patients found elevated effector cytokines (IL-1β, IL-6, IL-8, and MCP-1; IL-1β and TNF-α) compared to healthy controls (110). IL-6, IL-1β, and TNF-α are elevated in salivary sampling comparing active/exacerbated to inactive/remission CD patients, whereas active UC patients demonstrated increased IL-4, IL-10, and IL-21 (111, 112). Buccal mucosal sampling of pediatric CD patients revealed higher chemokines (CXCL-8, -9, -10) compared to healthy children and even adults with CD, suggesting possibly unique signatures of oral manifestations of pediatric IBD even when compared to adults (113). For one of the few studies that sampled the site of proposed inflammation, a study of gingival biopsies in adults with IBD and periodontitis revealed no differences between UC and CD but did show that across all sites, gingiva express IL-17A, IL-17F, IL-22, IL-25, IL-33, IL-10, and IFN-γ compared to the intestinal biopsies. These findings suggest that there may be unique inflammatory profiles in the oral niches compared to the intestine even at baseline, which establishes a challenging framework for studies interested in defining the local inflammatory profile of distant sites in the same subject (114).

### Microbial Dysbiosis

The first study to explore oral microbiomial shifts in IBD patients was by Van Dyke et al. who tied periodontal disease to oral dysbiosis. In this classic study, the oral microbiota was characterized in the periodontal pockets of two groups: 1) those with IBD and periodontitis and 2) those with IBD and only IBD-associated oral manifestations of soft tissues (115). The investigators found that the microbiota of IBD-associated periodontal pockets was unique compared to patients with IBD *but no oral involvement*., enriched with a unique microbiota of mostly small, gram-negative rods that were thought to be *Wolinella* (now: *Campylobacter*—a genus that is also associated with periodontal and other gastrointestinal diseases) (116, 117). Engel et al. focused on a case of severe, generalized periodontitis in a recent diagnosis of CD and also found a host of well-known periodontal pathogens in their patient, including *Porphyromonas gingivalis, Tannerella forsythia, and Campylobacter rectus* (84 *).*

Since gingival inflammation in IBD does not appear consistent with plaque accumulation, an interesting question is whether the systemic inflammation caused by IBD leads to changes of the subgingival microbiome, thus leading to dysbiosis that exacerbates oral inflammation. There is precedent for chronic inflammatory diseases like diabetes, systemic lupus erythematosus, or rheumatoid arthritis to influence the oral microbiome (118). Moreover, while mechanistically unclear, disturbances of salivary and oral mucosal microbiomes have been reported in two unique mouse models of colitis (DSS and *Citrobacter rodentium* infection) and this has been validated in humans (27, 119). The ability of IBD to induce inflammation due to the recognition of shared epitopes across the body has been suggested as a possible mechanism for extraintestinal manifestations of IBD (120, 121). Arguably, extraintestinal manifestations may be induced by local gut dysbiosis that causes a broad adaptive immune response that leading to the recognition of these epitopes in sites like the gingiva (121).

Building off of what had been thought of as dysbiosis in adult IBD and periodontitis patients, a 2012 study assessed the oral microbiome of the tongue and buccal mucosa in 114 healthy, pediatric IBD subjects using 16S rRNA profiling (122). This study found less overall oral microbiome diversity in CD compared to healthy controls—but not in UC. Tongue microbiomes revealed increased *Spirochetes*, *Bacteroides*, and *Synergistes* as well as decreased Firmicutes and Fusobacterium in IBD subjects compared to healthy controls. Another pediatric study sampled the subgingival microbial niche in 46 healthy and 35 CD patients (ages between 6 and 17 years old) before and after 8 weeks of pharmacotherapy (123). A majority of these cases displayed resolution of intestinal inflammation, but in treatment naïve CD patients, this study found increased *Capnocytophaga*, *Rothia*, and *Saccharibacteria* in the gingiva of IBD patients compared to healthy controls. When CD patients who had received antibiotic therapy were compared to a CD treatment-naïve cohort, the treatment cohort was shown to have decreased periopathogenic genera such as *Fusobacterium* and *Porphyromonas*, suggesting that some IBD treatments may have on reducing inflammation through changes to the oral microbiome.

Other IBD studies have found increased *Bacteroides*, *Prevotella*, and *Veillonella* and decreased *Proteobacteria, Neisseria, Gemella*, and *Haemophilus* when comparing the salivary microbiomes of IBD patients to healthy controls (110). A sampling of the subgingival microbiome in young and old patients have also shown unique oral dysbiotic signatures in IBD patients with gingivitis (increased *Prevotella*, *Peptostreptococcus*, *Streptococcus* species) or periodontitis (increased *Bacteroides*, *Campylobacter, and Porphyromonas species)* (124). A recent study of the salivary microbiome of UC and CD found increased diversity and enrichment of *Streptococcus* and *Enterobacteria* in UC and *Veillonella* in CD when either was compared to healthy controls (125). This group was able to identify distinct “oral ecotypes” for UC and CD; each was not defined by clinical characteristics or disease severity (125). The “indicator species” of these ecotypes varied over time but included *Corynebacterium* and *Acinetobacter* for UC and *Lactobacillus*, *Bifidobacterium*, *Scardovia*, *Streptococcus*, and *Pseudomonas* for CD. Ecotype 1 (CD) showed a specific enrichment of *Neisseria* and *Fusobacterium*. Furthermore, a recent study of the buccal mucosa in irritable bowel syndrome also revealed a decrease in Bacteroides and *Bacillus*, suggesting that gastrointestinal diseases other than IBD may influence the oral microbiome (126). Based on these studies, it appears that between pediatric and adult subjects, commonly increased taxa in untreated CD appear to include *Bacteroides, Campylobacter, Fusobacterium, Porphyromonas, Prevotella, and Veillonella*, which may provide important targets to better understand this gum–gut axis in future studies; however, the question of whether IBD-induced periodontal manifestations follow the same dysbiotic-immune paradigm as gingivitis and periodontitis remains unresolved.

## Defining the Gum–Gut Axis

### A Framework for the Understanding the Gum–Gut Axis

The decreasing cost and increasing sensitivity of high throughput assays to ask questions about host immunity across the life span make this an especially ripe time to understand the gum–gut axis in IBD. There is still a great deal to learn, but we believe that elucidating this axis may have important long-term ramifications for improving the health of oral and gastrointestinal tissues and decreasing disease incidence by guiding and training our host immune systems for a more balanced immune response later in life.

**Figure 4:**
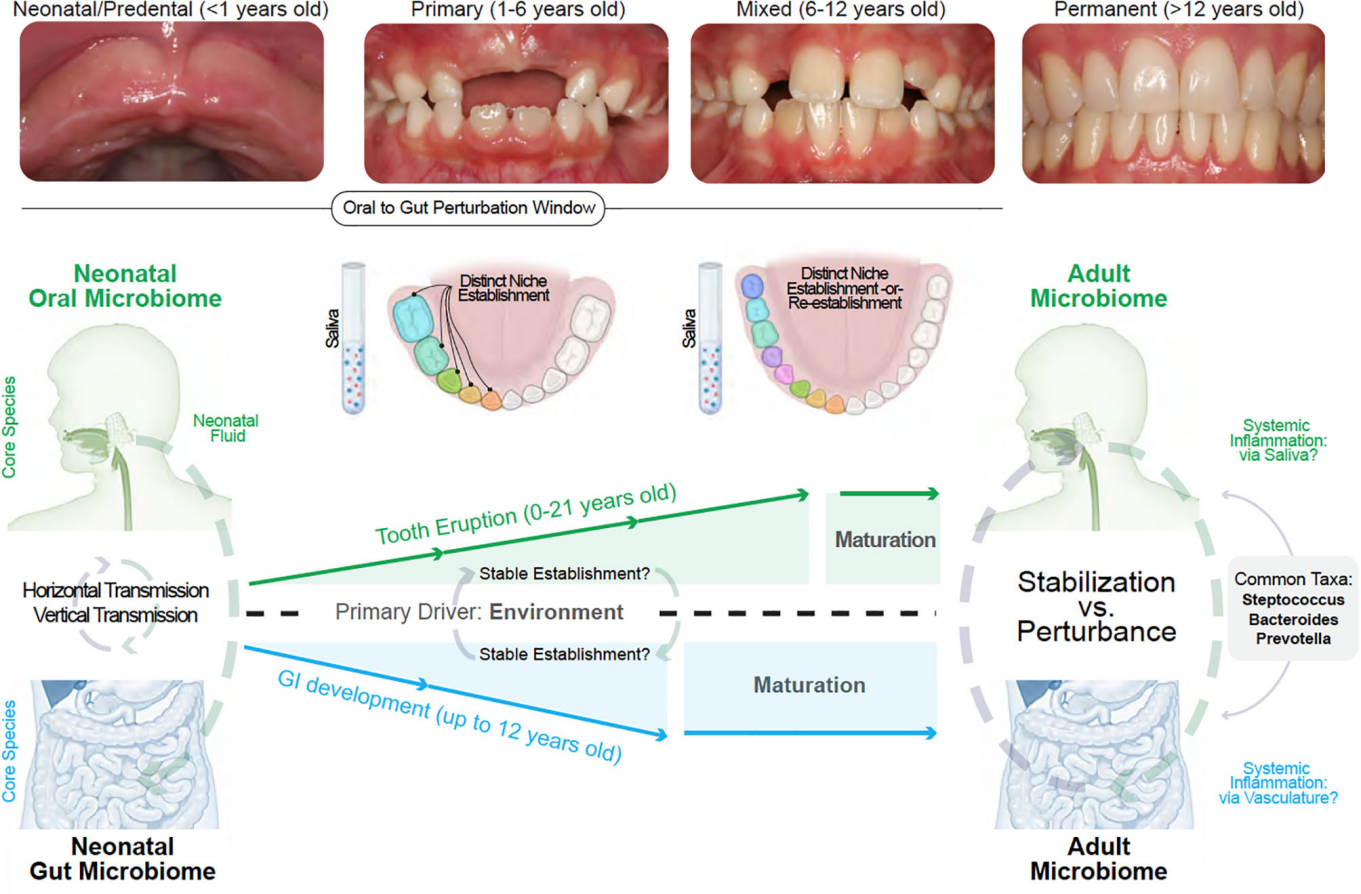
Linking the gum–gut axis from birth to adulthood. Due to the uniqueness of each site, neonatal and adult oral and gut microbiomes do not resemble one another. However, a common feature is that both sites and their distinct niches are established with a nascent microbiome that diversifies during development. The oral microbiome stabilize at a later development timeframe when compared to the gut microbiome due to the shedding of primary teeth until about 12 years old and the subsequent eruption of permanent teeth. This provides a narrower window of gut microbiome vulnerability compared to the oral cavity. Both oral and gut microbiome diversification and stabilization are reportedly driven by environmental influences. The influence of the oral cavity to the lower gastrointestinal tract can occur *via* saliva and also *via* vasculature (local inflammation seeding to distant sites) whereas the primary mode of gut to oral influence is *via* the mechanisms of local-to-systemic inflammation such as the delivery of effector cytokines or activation of oral tissue-resident immune cells.

These immune-microbiomial concepts are not new. For example, while preterm birth has been associated with dysregulated neonatal immunity, immune system “priming” in health is thought to occur through the birth canal from exposure to *Escherichia* and *Enterococcus* genera, as well as obligate/facultative anaerobes, including members of the Firmicutes and Bacteroidetes phyla and the *Bifidobacterium* genus. This narrow window for establishing long-term health is important for other mucosal tissues to help educate our mammalian immune systems (127). While immune priming has been recently shown to be important for the intestine through breastmilk (i.e., non-genetic inheritance of IgA), oral immune priming throughout neonatal oral microbiomial exposures remains to be fully explored (128). This will be even more important if early disturbances to oral immune priming are found to negatively affect gut immune priming (i.e., neonatal gum-to-gut influence; **Figure 4**).

We hypothesize that the uniqueness of the gingiva—from baseline immune profiles to the dynamic shifts of the microbiome—provides the possibility of connection to the gastrointestinal tract through the seeding of these products *via* gingival crevicular fluid and then *via* saliva. While a nascent field, the gum–gut axis also draws on a broad body of evidence. In the following sections, we will outline the interconnectedness of oral and gut microbiomial development and theorize how disturbances to these systems may influence disease. We will also emphasize saliva as a transmission vehicle for oral microbes and inflammatory cells/meditators to the lower gastrointestinal (**Figure 4**).

### The Interconnectedness of Oral and Gut Microbiomial Establishment

The human microbiome consists of a dynamic relationship between the microbiota—namely, viruses, protozoa, fungi, archaea, and bacteria—and their human niches; these niches can be found on the skin, the reproductive organs, as well as the interconnected gastrointestinal and upper aerodigestive tracts (including the lungs, intestines, and oral cavity) (129, 130). We are just now beginning to understand how this “forgotten organ” is established and matures along with the development of its host (131). The gut microbiome has been well-studied; however, historically, the oral cavity was one of the first sites used in the discovery of human microbes by Van Leeuwenhoek over three centuries ago (132).

Currently, it is unknown exactly how many microbial species exist in the pediatric or adult human oral cavity, but the Human Oral Microbiome Database (http://homd.org/) lists over >150 genera, 700 species, and 1,300 strains, including an incredibly diverse mycobiome of almost 100 fungi genera, and nearly 5,000 biosynthetic gene clusters which produce small molecules to allow for microbial communication (133–136). The vast majority of oral microbiota are commensals—but also include symbiotes and pathogens—and as more strains are discovered, the more it is recognized how microbial diversity and, importantly, their interdependence is dynamic (137). Unsurprisingly, comparing adult oral and gut microbiomes reveals that they are more dissimilar than similar; however, there are some common taxa between the two, including *Streptococcus*, *Bacteroides*, and *Prevotella*. The relative abundance of *Streptococcus* and *Bacteroides* differ significantly, though *Prevotella* was found to be closely matched when considering a grouping of buccal mucosa and hard palate versus a stool sample (~3% abundance in both groups) (138, 139). Considering another grouping of the tongue, salivary, and oropharyngeal samples resulted in 4× fold relative detection of *Prevotella* compared to other oral and GI microbiomes; consistently, however, there is substantial heterogeneity of oral microbiomes when comparing niches core species.

Relevant to the gum–gut axis is how the oral and gut microbiomes develop and how these unique microbiomes evolve as we age (140). It is now thought that oral and gut colonization between living partners and between those partners**’** children is the direct influence of both horizontal and vertical transmission mechanisms, respectively (141–144). Oral microbiomes cluster more clearly when age and niche are considered; this is an important point to consider moving forward when thinking about oral–gut association studies (145). While the stable establishment of the host microbiomes has been shown to influence the health of the individual later in their life (146–148), whether this establishment occurs *in utero* remains controversial (149, 150). Currently, it is thought that, while *in utero* colonization may be possible, it is also restricted. Recent 16S studies of meconium have detected only 18 total taxa assumed to be from the gut, dominated by *Micrococcus* and *Lactobacillus* (151). How this relates to future oral and gut mucosal immunity remains to be discovered.

Though ecologically distinct and with their characteristic species, the oral and gut microbiomes both become more diverse after birth; however, the gut microbiome stabilizes earlier than the oral microbiome. This is likely due to the longer period of growth, development, and tooth eruption in the oral cavity in the first two decades of life (152, 153). This trend also follows for the intestinal immune system compared to the oral cavity (154). When considering the gum–gut axis, we do not currently know if and when “perturbation windows” to either microbiome may more effectively compromise the health of the oral and the intestinal microenvironment (**Figure 4**).

The establishment of stable oral microbiomes may also be critical for overall health in children; for example, *Bifidobacteria* are known to be an important component of a healthy gut microbiome—especially during lactation and breastfeeding—and have been shown to influence immune responses in the gut (155). Interestingly, recent work has shown that *Bifidobacteria* may predominantly seed the gut *via* neonatal oral fluid (156, 157). Another important question is how breastfeeding, which is filtered through the mouth, may simultaneously influence the development of both oral and gut microbiomes. For example, the neonatal gut virome has been shown to develop sequentially through breastfeeding (158), and it is known that the oral cavity can host several unique viromes that likely benefit from this mode of neonatal feeding (159).

### Oral Microbiomial Development: Oral Mucosa, Subgingiva, and Saliva

To connect the gum to the gut, the goal of this section is to establish a logical link between predentate mucosal microbiomes, the subsequent emergence of teeth to establish the first subgingival microbiomes, and then to connect this site to the lower gastrointestinal tract through the oral biofluids around the tooth (via gingival crevicular fluid) and then the whole oral cavity (via saliva). We hypothesize that chronic disruption of the oral immune repertoire sets up a gum–gut axis whereby the gingival microbiota shift towards dysbiosis, establishing a positive feedback loop for a chronic lesion of effector immune cells that can travel and occasionally survive the journey in saliva to the rest of the body, including the lower gastrointestinal tract (**Figure 4**).

While the oral microbiome is often referred to as an individual entity, it is important to emphasize that there are several ways to think about the microbiomial niches within the oral cavity. For example, it is known that there is a 1) biogeographical (i.e., spatial) diversity of species within polymicrobial communities (160), 2) biofluidic diversity comparing salivary to the gingival crevicular fluid, 3) niche-by-niche diversity between oral mucosal sites (161), and even 4) anteroposterior diversity proposed to be caused by retrograde salivary flow during swallowing (162). Despite this vast heterogeneity, most single niches only contain about 5% of the total known species (137). Some species are common to multiple sites, each with a preference for various “landscape ecologies” of the oral cavity that support this niche heterogeneity over time (163). Recent work considering the site-specificity of adult oral microbiomes has documented these unique species for the dorsal tongue mucosa, gingiva, and the enamel surfaces of teeth (161).

There is also the question of temporal heterogeneity. Our oral and gut microbiomes continually change throughout our life, but even the most primitive microbiomes in neonates are now thought to play an active role in our oral and gut development (164). While host genetics may contribute to microbiomial development in a niche, a recent twin study of oral microbiomial variation of the supragingival niche over 12 months suggests that environmental exposure may be the primary influence on microbes found in each niche (165). This has also been shown for salivary microbiomes in a cohort of children between 2 days and 5 years old (166). The development of microbiomes in the oral cavity is highly unique compared to the rest of the body; this is because, in children and adolescence, these microbiomes are often defined by the dentition “state”. In neonates, this is often staged as a mouth with no teeth present (neonatal/predentate mucosa); toddlers display primary dentition; children display a mixed dentition, and teenagers display a permanent dentition with only adult teeth (**Figure 4**).

Temporally, predentate neonates display the most rudimentary oral mucosal microbiomes, but even at this timepoint, 50 genera have been identified, the majority of which appear to be facultative anaerobes and anaerobes (167). Primary colonizers (0–3 months old) include *Lactobacillus, Fusobacterium, Staphylococcus, Streptococcus*, and *Veillonella*; secondary colonizers include *Gemella, Granulicatella, Haemophilus*, and *Rothia.* Neonatal microbiomes also appear to vary at the species-level with only ~30 “core species” identified (167). At 3 months old, which is still before the first teeth erupt for most infants, predominant oral microbiomial phyla include *Saccharibacteria* (formerly *TM7)*, *Fusobacteria*, and *Actinobacteria*—but not in all children, again supporting the importance of environmental influence on microbiome development (168). Oral mucosal microbiomes have been shown to increase in diversity and richness along with the developmental progression of predentate to permanent (145). It has been reported that the neonatal oral mucosa displays a high microbiomial diversity but low richness (169). Predentate microbiomes are populated with *Bacteroides*, Firmicutes, *Eikenella, Eubacterium, Gemella*, *Granulicatella, Oribacterium, Proteobacteria, Selenomonas*, *Streptococcus*, and *Veillonella*.

It is on the foundation of this predentate mucosal microbiome that the subgingival microbiome is established, and there is a correlation between maternal and neonatal predentate core species. This is critical and sensitive. When mothers who were also smokers were studied (passive maternal smoking), an increase in periodontal pathogens such as *Campylobacter* and Fusobacterium were uniquely noted in the predentate microbiomes of their offspring. These are important to emphasize because they are also known IBD pathobionts, and a recent multicenter study found that passive maternal smoking had a dose-dependent association with the development of pediatric IBD (170). Additionally, maternal smoking habits in the perinatal period have been associated with developing IBD in their offspring (odds ratios of 5.32 for CD; 3.02 for UC) when matched to unexposed controls, and we hypothesize that these oral microbial alterations may play an active role in the exacerbation or acceleration of IBD.

Once the predentate microbiome is established, the subgingival microbiome forms as teeth erupt through that oral mucosa to form the subgingival crevice. This niche is unique in the mouth, protecting from oxygen and low redox potential for Gram-negative anaerobes (96, 171). Studies focused solely on the gingival pocket have detected nearly 500 different species in the subgingival biofilms attached to the tooth and with few species dominating in adults (172). This unique anatomy results in a complex microbiota even in children, with only about 1/3 of genera shared with the predentate microbiomes (167). In this niche, several taxa are common to primary, mixed, and permanent dentition states, including *Actinomyces*, *Capnocytophaga*, *Campylobacter*, *Corynebacterium*, Fusobacterium, *Gemella*, *Granulicatella, Haemophilus. Kingella, Porphyromonas, Prevotella, Streptococcus, Terrahaemophilus*, and *Veillonella* (167).

As permanent teeth erupt, the richness of species increases as does oral microbiomial “personalization”. This is the site of microbiomial dysbiosis in gingivitis that can occur in children and adults. In both classical and IBD-associated gingivitis, the gingival crevicular fluid flows outward through the junctional epithelial attachment into the subgingival crevice and eventually the saliva. It is thought that this crevicular fluid may also serve as nutrition for the oral microbiome. As more species colonize the subgingival space, gingival crevicular flow rates and pH have been shown to increase (173, 174). A few recent studies on adults have assessed this fluid for its microbiome; however, because it flows into saliva and likely contains free-floating microbes from the developing subgingival biofilms, its signature appears more as an intermediate between these two niches (175–177).

Changes to the salivary microbiome during oral development have also been examined (167). Saliva is not solely a microbiome source but also contains factors that support commensal microbiomial development and maintenance (178). Like other oral microbiomes, the salivary microbiomial composition is also defined by ecological succession (168). The salivary microbiome has very few “core species” shared between these developmental stages (167); however, the concept of “early colonizers” has been explored and suggests that *Streptococcus* and *Veillonella* appear first, followed by *Neisseria.* What appears most consistent is that salivary microbiomes are closely aligned with the dentition state; thus, the salivary microbiome clusters with the predentate microbiome in neonates and clusters more with the primary teeth stage microbiomes in toddlers, etc. This results in salivary microbiomes that increase in diversity as primary teeth first erupt, are shed, and permanent teeth again erupt. For example, neonatal salivary taxa are dominated by *Streptococcus, Veillonella, and Gemella*—much like the neonatal microbiome. When the primary dentition erupts, similarities to the maternal oral microbiomes become less apparent, which accompanies the detection of *Actinomyces*, *Corynebacterium, Granulicatella*, Fusobacterium, *Haemophilus, Neisseria*, and *Rothia* (167). This reflects the ability of saliva to be sampled as a gestalt—but not as a specific— readout.

### Linking the Oral Microenvironment to the Gut Through Saliva

Currently, there are several proposed mechanisms for how periodontal diseases may influence distant sites like the intestines. These links include dissemination of periopathogens and inflammatory mediators like TNF, IL-1β, and IL-6 systemically through the bloodstream (179) Though many studies have suggested a potential for chronic periodontal inflammation and local oral dysbiosis to influence other body sites, few studies have determined causality (i.e., whether this is occurring *via* the distant effects of inflammation from chronic gingivitis/periodontitis or transmission of oral pathogens to distant sites). The latter considers a role for translocation of the subgingival microbiome; however, one group recently examined the gut microbiomes of patients with health, gingivitis, and periodontitis. They found that chronic oral inflammation was associated with less alpha-diversity in the gut microbiome, and though some oral taxa could be detected from the stool of each patient cohort, no clear trends for oral taxa enrichment emerged in this pilot—thus pointing to the possible influence of gut microbiome composition through oral inflammation (180).

From the recent literature, the gum–gut axis appears to be inherently linked through saliva, which can deliver enzymes, effector cytokines, free-floating and keratinocyte-bound bacteria, and subpopulations of viable inflammatory cells such as neutrophils, lymphocytes, and macrophages to distant sites. Saliva also contains mucus (comprised of water, lipids, and proteins such as mucins) which can protect these contents from the acidic contents of the stomach for survival along the gastrointestinal tract (181). About ~1 to 1.5 L of saliva is produced daily per person, and this contains millions of bacteria that are traditionally not known to colonize distant intestinal sites in health (182). While there is an interest in whether and how dysbiotic subgingival microbiomes could lead to the subsequent release of pro-inflammatory cytokines, this is a burgeoning field of study. Recent studies in mice showed a role for known periopathogen *Porphyromonas gingivalis* in perpetuating systemic inflammation after oral administration in mice; this led to endotoxemia, altered the gut microbiome, decreased insulin resistance, and altered tight junction expression in the ileum (183). Other studies have linked *Atopobium parvulum, Campylobacter concisus, Fusobacterium nucleatum, Fusobacterium varium*, and *Staphylococcus aureus* to gastrointestinal disease, but whether these species are colonizing the intestine or indirectly eliciting chronic immune responses remains to be seen (184).

Several interesting studies have put forth the first evidence of transmission and colonization of oral microbes to the upper aerodigestive tract and also to the intestine (gum to gut influence). In health, there is evidence for oral microbiomial contribution to the oropharyngeal, esophagus, and gastric microbiomes (185). For example, an early study of the distal esophagus found 13 genera common to all samples, including *Streptococcus*, *Prevotella*, and *Veillonella*; most species-level OTUs were determined to be similar or identical to those of the oral cavity (186). This distant transmission is not specific to the GI tract as colonization and succession of the lung microbiome are associated with cystic fibrosis progression in infants and children (187). This work showed colonization of the lungs by oral microbes was possible even in 2-year-old children, with a significant abundance of *Streptococcus*, *Prevotella*, and *Veillonella*—identical to those found in the distal esophagus. Periopathogen taxa such as Fusobacterium and *Porphyromonas* were detected as well in some progressing groups, supporting the potential role for known periopathogens transmission in disease progression in a distant site.

This transmission phenomenon is also observed in the lower gastrointestinal tract (188, 189). Given these findings, what was once thought to be a rare event—the colonizing of distant microbiomial niches by oral microbes—appears to be more commonplace than once appreciated. For example, recent work assessing both salivary and stool samples primarily from healthy adults estimated that >10% of oral species may transmit *via* an oral-fecal route throughout the entire GI tract (190). This demonstrates a previously underappreciated niche-to-niche colonization pipeline. Other studies have reported many oral microbes found in the intestinal tissues of adult patients suffering from IBD, such as *Aggregatibacter, Campylobacter, Enterobacteria*, Fusobacterium*, Gemella, Neisseria, Pasteurella, Peptostreptococcus*, and *Streptococcus*. Many of these are associated with gingivitis (184). These findings are supported by well-designed studies in mice that have demonstrated competition for the GI niche by oral and traditional gut microbes (191), as well as other recent work that has demonstrated the establishment of oral microbes in the gastrointestinal tracts of patients afflicted with colorectal cancers and adult IBD (185, 188, 192, 193). A recent study of IBD found colon biopsies were abundantly colonized by periopathogens such as Fusobacterium*, Peptostreptococcus, Staphylococcus*, and *Streptococcus* (194).

Mechanistic studies using gnotobiotic mice have also shown a role for resident *Klebsiella* spp. in the saliva of IBD patients to colonize an already dysbiotic colon, leading to a significant inflammatory response through Type 1 T helper (TH1) cells in the local gut microenvironment (28). Whether *Klebsiella* spp. are truly a pathogenic link between the oral cavity and the gut or merely a demonstration of pathogenic colonization and immune-mediated exacerbation of IBD in mice remains to be elucidated. However, there is an exciting future ahead, especially when considering a recent study that utilized a ligature model in mice to induce periodontal inflammation. This led to subgingival dysbiosis with increased *Bacteroides*, *Enterobacteriaceae*, and *Staphylococcus*. *Enterobacteriaceae* were also found in the gut microbiome, again suggesting that oral microbes were able to colonize the intestine, and also exacerbate, established colitis. This study found that this was dived *via* oral niche primed Th17 cells with tropism for the gut (29). While these studies have not yet answered how these systems are explicitly linked, there is increasingly strong evidence for oral dysbiosis and localized gingival/periodontal inflammation eliciting and exacerbating and immune responses in the gut. While much of this review has focused on the oral gingival niche as one half of the axis (gut to gut inflammation and subsequent gut to gum influence), this reflects the state of the field. The local gut niche in IBD with and without chronic oral inflammation is a nascent field with many more mechanistic and clinical studies needed.

## Discussion

**Figure 5:**
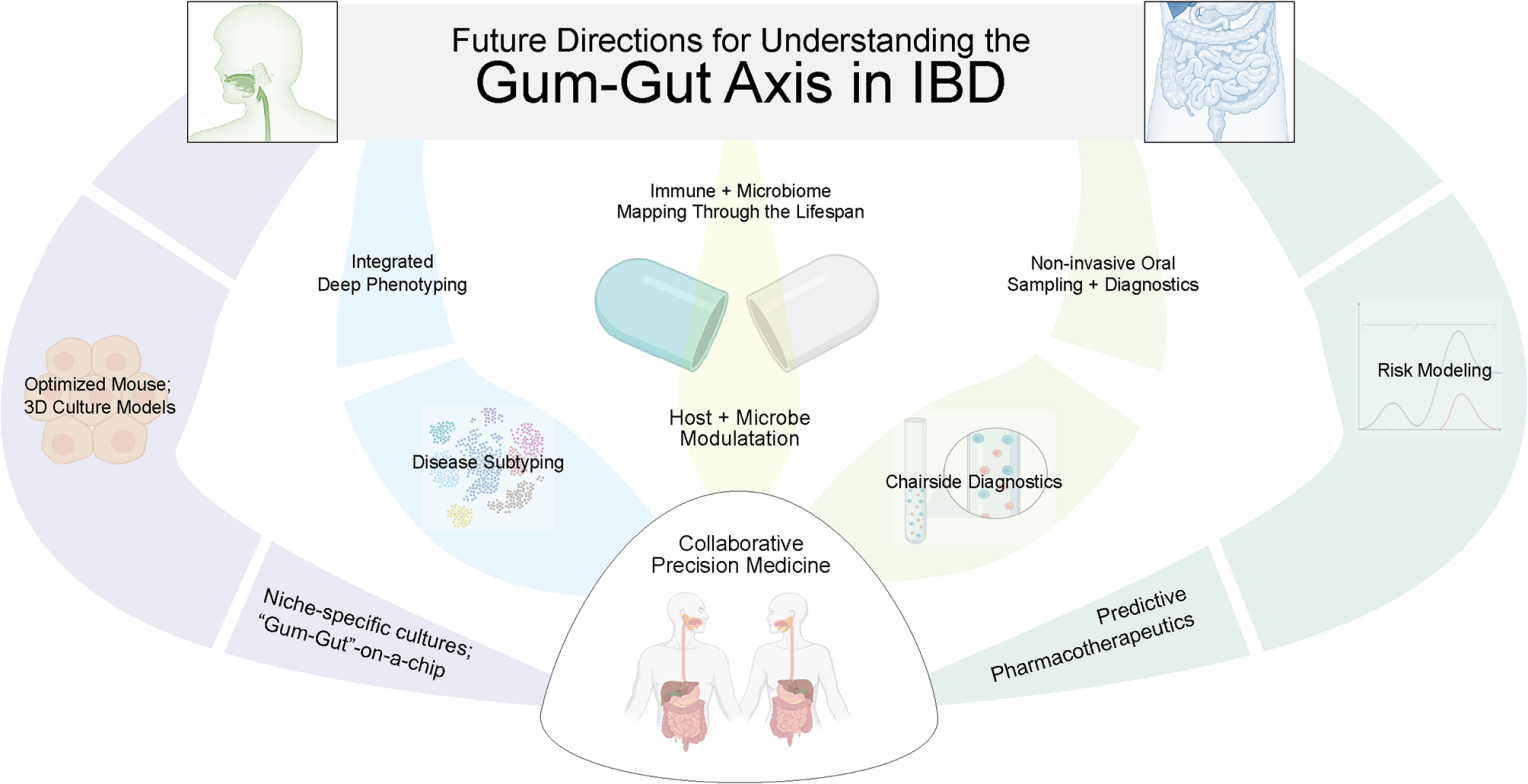
Future directions to better elucidate the gum–gut axis. Due to the uniquely active and remittent inflammatory states of periodontal disease and inflammatory bowel disease, there is an exciting future for collaborative efforts between GI and oral health care providers to answer these questions. This will require biosampling both oral and intestinal sites to correlate dysbiosis and inflammatory changes across niches and across time, better modeling using *ex vivo* models, and better phenotyping of both diseases. In the future, predictive modeling and precision medical approaches are possible to treat both oral and GI diseases.

There remain several challenges to formally establishing our proposed gum–gut axis. In particular, the heterogeneity of IBD and periodontal disease—as well as the temporal nature of each to exhibiting periods of activation and remission—make this association difficult to establish until better subtyping and disease activity for each disease can be more clearly ascribed (**Figure 5**). It remains critical that we continue to learn more about the pathogenesis of both oral and GI diseases so that we can also better understand how they influence each other. This will allow for the development of future tools to improve both our oral and gastrointestinal health throughout life. Additionally, while oral manifestations are not often the primary concern for treatment in many systemic diseases like IBD, their oral manifestations may provide a “window to the rest of the body”, serving as an easily accessible site to aid in the diagnosis or to serve as a functional readout of disease activity (195). This could include frequent oral sampling through treatment naïve IBD patients to predict disease activity or to determine the efficacy of IBD biologics/biosimilars chairside before and during the critical first months after deciding on a particular IBD pharmacotherapeutic.

In parallel with a non-invasive sampling of the oral cavity in longitudinal studies, it is clear that model organisms such as mice are proving vital to provide supporting evidence for the gum–gut axis. This type of work is supported by a recent resource of oral microbiomial development that has cataloged predentate, eruption, and post-eruption stages in mice. This provides a framework for studies about niche establishment, dysbiosis, and the long-term consequences and resistance to gum–gut disease. Additionally, ileitis models (such as SAMP1/YitFc mice) and DSS models of colitis are reported to show oral mucosal inflammation and inflammatory bone loss that mimics periodontitis in mice and could be useful for these investigations (196, 197). While it is interesting to postulate about which oral taxa could take residence and cause, reactivate, or exacerbate IBD, further studies are required to understand how gut microbial growth rates, antibiotic resistance (i.e., resistomes), microbial gene expression, and metabolomics all come together to influence IBD development and perpetuate IBD (198–201).

While much work remains to be done, there is an exciting future for collaborative efforts between GI and oral health care providers to answer these questions. This will require biosampling both oral and intestinal sites to correlate dysbiosis and inflammatory changes across niches. It is encouraging to see the progress of the NIH Human Microbiome Project over the last decade that originally collected nasal, oral, gut, skin, and vaginal microbiomes from a healthy cohort and provided valuable resources such as the Human Microbiome Project Data Coordination Center (202). As we begin to understand both the window of stable microbiomial establishment for the oral and gut microbiomes, we may be able to intervene to impact the health of these two sites for improved health later in life. For example, there are already several active clinical trials related to reestablishing a healthy neonatal gut microbiome (131). To justify the clinical implementation of this type of intervention will require *in vitro* 3D modeling (203), pre-clinical animal studies of host-microbiome interactions (204–207), interdisciplinary clinical research projects focused on longitudinal biobanking (208) for multiomics-informed approaches (199, 209, 210), biomarker discovery and validation, and improved risk modeling (211, 212). Ultimately, such work will lead to personalized therapeutics to target the **gum–gut** axis (213), sensitive and specific tools for early diagnosis of diseases in each site, and the exciting possibility of new biomarkers for risk stratifying a spectrum of oral and gastrointestinal diseases (214, 215).

## Author Contributions

Conceptualization: KB and AG. Investigation and data analysis: KB. Writing the original draft: KB. Writing, review and editing: KB and AG. All authors contributed to the article and approved the submitted version.

## Funding

This work was supported by NIH grants to KB (NIDCR K08 DE026537) and to AG (NIDDK P30 DK034987, PI: Robert Sandler).

## Conflict of Interest

The authors declare that the research was conducted in the absence of any commercial or financial relationships that could be construed as a potential conflict of interest.
